# Narrowband random lasing in a Bismuth-doped active fiber

**DOI:** 10.1038/srep30083

**Published:** 2016-07-20

**Authors:** Ivan A. Lobach, Sergey I. Kablukov, Mikhail I. Skvortsov, Evgeniy V. Podivilov, Mikhail A. Melkumov, Sergey A. Babin, Evgeny M. Dianov

**Affiliations:** 1 Institute of Automation and Electrometry SB RAS, Novosibirsk, 630090, Russia; 2 Novosibirsk State University, Novosibirsk, 630090, Russia; 3 Fiber Optics Research Center RAS, Moscow, Russia

## Abstract

Random fiber lasers operating via the Rayleigh scattering (RS) feedback attract now a great deal of attention as they generate a high-quality unidirectional laser beam with the efficiency and performance comparable and even exceeding those of fiber lasers with conventional cavities. Similar to other random lasers, both amplification and random scattering are distributed here along the laser medium being usually represented by a kilometers-long passive fiber with Raman gain. However, it is hardly possible to utilize normal gain in conventional active fibers as they are usually short and RS is negligible. Here we report on the first demonstration of the RS-based random lasing in an active fiber. This became possible due to the implementation of a new Bi-doped fiber with an increased amplification length and RS coefficient. The realized Bi-fiber random laser generates in a specific spectral region (1.42 μm) exhibiting unique features, in particular, a much narrower linewidth than that in conventional cavity of the same length, in agreement with the developed theory. Lasers of this type have a great potential for applications as Bi-doped fibers with different host compositions enable laser operation in an extremely broad range of wavelengths, 1.15–1.78 μm.

Physics of light interaction with amplifying disordered optical media attracts a great deal of interest as a fascinating interdisciplinary field connecting many branches of science and generating new results of fundamental and applied significance. Random lasers (RLs), in which a conventional optical cavity is substituted with a multiple-scattering feedback in a disordered amplifying medium, represent now a rapidly growing class of light sources, see^1^,^2^,^3^ for a review. In early works traditional active media such as laser crystals or semiconductors, processed to a powder or granular state, were explored. Recent developments in this field are focused on the demonstration of lasing in random media of new types. In particular, not only the normal gain in active media, but also the Raman amplification in passive media is explored^4^,^5^. As shown in^5^, high-power non-resonant pumping enables the Raman lasing in non-active random materials (such as BaSO_4_), which makes random lasing possible in almost any “white” powder, thus offering a new diagnostic tool for turbid/granular media with a great potential in remote sensing and pharmacology.

Another important direction is the development of new RL-based light sources, e.g. low-coherence sources suitable for speckle-free full-field microscopy or digital light projector systems^6^. For this and similar applications a competitive performance of the light source becomes a challenge. In this sense, fiber-based random Raman lasers^4^,^7^ are among promising candidates, because their output characteristics are superior to random lasers of other types, and in some cases to conventional lasers. The fiber waveguide structure is nearly one-dimensional forming an output beam of high quality (single transverse mode with a Gaussian beam profile) in a desired direction controlled via fiber bending. For random lasing, even conventional telecom passive fibers are suitable. As the fiber material (silica glass) is highly transparent to radiation, especially in the telecom spectral window near 1.55 μm, the gain and feedback mechanisms here are quite different from those in other random lasers. The fiber gain is induced by inelastic stimulated Raman scattering (SRS) of the pump light by vibrating SiO_2_ molecules in a glass lattice, whereas the feedback is provided by elastic Rayleigh scattering (RS) of the SRS-induced Stokes wave on sub-micron irregularities of the glass structure, with a small part (~10^−3^) of scattered light coming back into the fiber. Though the feedback is weak, it is sufficient for lasing in a kilometers-long passive fiber given that the integral Raman gain (proportional to the fiber length and pump power) compensates for the roundtrip losses.

Fiber-based random Raman lasers demonstrate at the moment the highest optical efficiency of pump-to-Stokes wave conversion exceeding 87% already at Watts-level powers^8^, while the maximum output beam power reaches 200 W^9^. Such random Raman fiber lasers (RRFLs) generate a quasi-continuous mode-free spectrum with a resulting shape defined by the Schawlow-Townes narrowing near the threshold and nonlinear broadening at high powers^7^,^10^. Fiber-based spectral filters can be relatively simply imbedded into the low-power part of RRFLs providing flat tuning within the entire Raman gain spectral range of >35 nm^11^, as well as power-equalized multi-wavelength generation^12^, and order-of-magnitude spectral width reduction defined by the filter characteristics^13^. Other RRFL features improving their performance are being also developed, e.g. linear polarization of the output beam^8^, direct pumping by high-power LDs^14^, short-pulse generation with wide spectral and temporal tuning^15^, cascaded generation of high-order Stokes waves^16^,^17^, etc. (see ref. 7 for a review). Hybrid schemes employing amplification in a short active fiber doped by rare-earth elements (Er, Yb) and the RS feedback in a separate long passive fiber are also studied^18^,^19^, but they are closer to conventional lasers than to random ones. The only common feature with random lasers is modeless spectrum free of mode competition which results, e.g., in a flat tuning curve, just like in random RFLs^11^. Another way for random lasing in active fibers is to inscribe into it a randomized highly reflecting fiber Bragg grating^20^, but its characteristics are closer to regular distributed-feedback laser generating single-frequency radiation.

Here we report on the first demonstration of a random laser based on an active fiber with the RS feedback. This became possible due to the implementation of a new Bismuth doped fiber with an increased amplification length and RS coefficient. The Bi-doped fiber random laser generates in a specific spectral region (1.42 μm) exhibiting unique features, such as an ultra-narrow bandwidth as compared with a conventional-cavity Bi-fiber laser of the same length and output power. A theory describing the spectral broadening in fiber lasers of different types (with a conventional linear cavity or random distributed feedback) in a general way explains the fundamental difference in their spectral broadening induced by self-phase modulation. It is shown that the much weaker broadening in the Bi-fiber laser with random distributed feedback at the same output power level is reasoned by a much lower nonlinear phase incursion caused by a principally different longitudinal power distribution.

## Results

### Experiment

**Figure 1:**
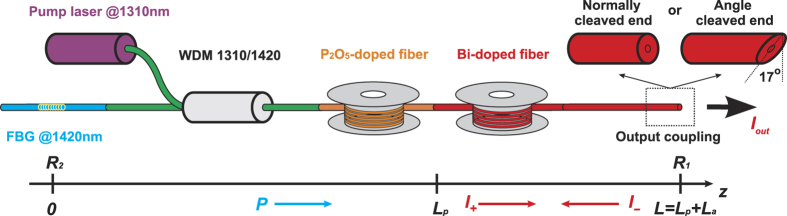
Scheme of Bi-doped fiber laser: 1310-nm laser pumps via a WDM Bi-doped fiber (BDF) that becomes lasing in the half-open cavity formed by the FBG and random distributed feedback via the Rayleigh scattering (RS) in the angle (17°) cleaved fiber. A passive P_2_O_5_-doped fiber (PF) and 4% reflection from the normally cleaved end forming the linear cavity were added to perform comparative studies.

The random laser based on an active Bi-doped fiber (BDF) is schematically shown in Fig. 1. The BDF (manufactured by FORC^21^) with a length of 150 m is used as an amplifying/scattering medium. The integral reflectance of the fiber due to the RS is measured to be 3.8·10^−6^; nevertheless, the high gain coefficient of the active fiber at the wavelength of 1420 nm appears to be enough for lasing even with such a week feedback. The gain is achieved by pumping the fiber through the 1310/1420 wavelength-division multiplexer (WDM) by a home-made Raman fiber laser with the maximum output power of 4.4 W at 1310 nm corresponding to the 1^st^ Stokes component of a phosphosilicate fiber pumped by a 1115-nm Yb-doped fiber laser^22^. The wavelength of pump radiation lies in the absorption band of Bismuth active centers in silica-based fibers^21^. The output end of BDF is cleaved with an angle of ~17° in order to avoid the Fresnel reflection. The reflection coefficient from the output end is measured to be −65 dB, so the feedback in the cavity is mainly provided by the random Rayleigh scattering distributed along the fiber. At the other BDF end, a highly-reflective fiber Bragg grating (FBG) is inserted to form a half-open cavity with spectral filtering at each round trip. In the experiments, two FBGs with the Gaussian reflection spectrum at 1420 nm are explored: wideband FBG with a reflection bandwidth (FWHM) of 1.88 nm and narrowband FBG with 0.28-nm FWHM. Their reflection coefficients are 50% and 70%, respectively.

**Figure 2:**
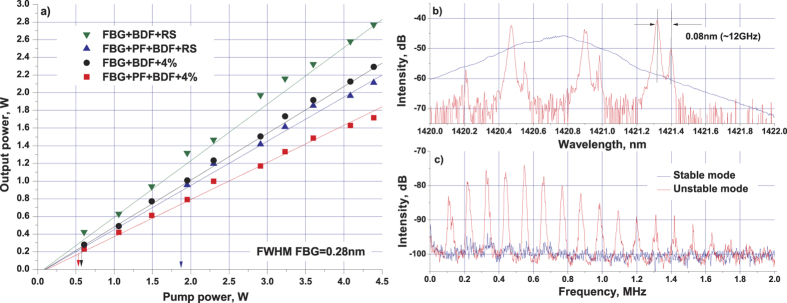
Bi-doped fiber laser characteristics: Output power as a function of the pump power for different cavity configurations, the arrows indicate the stability thresholds (**a**). The corresponding optical (**b**) and RF (**c**) spectra above and below the stability threshold. The results are presented for the schemes with a narrowband FBG (FWHM = 0.28 nm), similar dependences are observed for the schemes with a wideband FBG (FWHM = 1.88 nm).

The first experiments with the 150-m BDF showed that its random lasing threshold is exceeded already at 0.5 W pump power (see Fig. 2a), but the generated spectrum is unstable. The instability manifests itself in a narrowband double peak structure appearing randomly around the central wavelength of 1420.7 nm (see Fig. 2b). The frequency spacing between the peaks is constant amounting to ~12 GHz, which corresponds to the Brillouin shift in the fiber. It is well known that the cooperative Rayleigh-Brillouin scattering^23^ is the main reason for such instability in RS-based random fiber lasers operating near the threshold, which could be suppressed far above the generation threshold^4^,^7^. Nevertheless, an increase in the pump power in our case has not led to stabilization of the Bi-doped fiber laser spectrum, while its output power grows nearly linearly reaching 2.8 W at the maximum pumping (corresponding efficiency amounts to 64%). At that, the generated Bi-related line at 1420 nm is well separated from the negligibly small SiO_2_-related Raman line at ~1400 nm.

It was found that stabilization of the generated spectrum is achieved by means of cavity lengthening with a passive fiber (SMF-28e, for example) placed before the Bi-doped active fiber (i.e. FBG + PF + BDF). The stability threshold decreases with increasing total cavity length. This fact can be associated with suppression of the Brillouin scattering due to the spectral broadening of generated peaks in a longer fiber. For passive fibers of equal length, the lowest threshold is observed for the phosphosilicate (P_2_O_5_-doped silica) fiber, probably because of the different Brillouin shift taking that the Rayleigh scattering coefficients for used passive fibers were approximately equal. In further experiments, an 800-m phosphosilicate fiber (PF) spool was inserted into the scheme for laser stabilization (see Fig. 1). Testing of the scheme in which active and passive fibers are placed in opposite order (i.e. FBG + BDF + PF) has shown that there is no stable laser generation in this case.

The total reflectance of all fibers due to the RS is measured to be 10^−5^ at the laser wavelength of 1420 nm. It should be noted than there is no lasing at 1420 nm in the absence of BDF (i.e. FBG + PF) even at the maximum pumping. In the combined PF-BDF half-open cavity the instability is observed below 2 W (see arrow marks in Fig. 2a) manifesting itself similarly to the scheme without PF: The Brillouin peaks in the spectrum (Fig. 2b) are accompanied by high-peak pulsations of intensity with appearance of a longitudinal mode structure in the radio frequency (RF) spectrum (Fig. 2c). The intensity dynamics and RF spectra were analyzed by a 2.5 GHz-bandwidth oscilloscope (LeCroy WavePro725ZiA with a spectrum analyzer option). In the stable domain, the generated spectrum becomes smooth and broadened (Fig. 2b, solid curve), the intensity dynamics becomes quasi-continuous and the mode structure in the RF spectrum disappears (Fig. 2c). The last fact confirms the major role of the RS in the lasing process, which becomes dominating over reflection from the angled cleaved fiber end in the elongated cavity, while in the short BDF cavity the end reflection may have some influence. From the obtained data we estimate the stability threshold in 150-m BDF as ~10 W.

**Figure 3:**
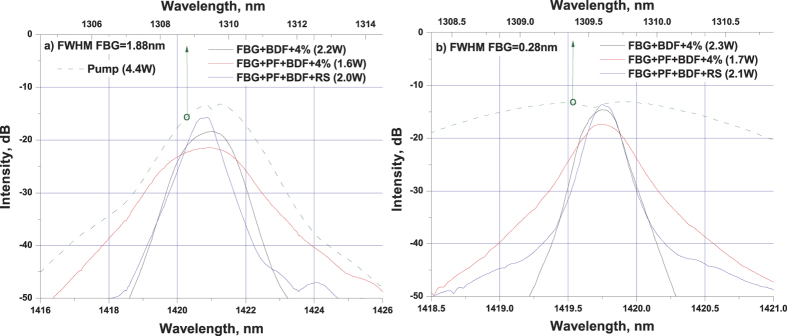
Generation spectra for stable laser schemes with broadband (**a**) and narrowband (**b**) FBGs at the maximum pump power of 4.4 W (the output power is indicated for each scheme). The pump radiation spectra are also shown being related to the upper axis.

**Figure 4:**
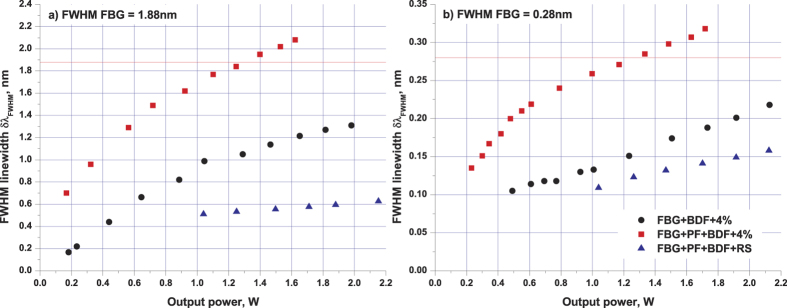
Power evolution of lasing linewidth (FWHM) for stable laser schemes with broadband (**a**) and narrowband (**b**) FBGs.

The maximum output power in the scheme with PF is slightly (by ~20%) lower than that in the pure BDF random laser, but it remains to be above 2 W at 4.4 W pumping with the conversion efficiency of about 50%. The lower efficiency is explained by the PF-induced additional losses. For comparison with traditional lasers, two BDF laser schemes (with and without the PF) with the conventional cavity based on a lumped output reflector are also investigated. For this purpose, the angle cleaved fiber end was replaced by the normally cleaved one with the Fresnel reflection of ~4% (Fig. 1). In this case, the output power further decreases by 20% (Fig. 2a) in comparison with the corresponding RS-based scheme. Thus, the RS-based PF-BDF scheme has similar output characteristics to the 4% cavity with the BDF only, see Fig. 2a. Despite this similarity, their output spectra are considerably different. The generation spectra at the maximum pump power for random and traditional laser schemes with broadband and narrowband FBGs are presented in Fig. 3a,b, respectively. The comparison shows that the narrowest linewidth is obtained for the PF-BDF scheme with the RS and the broadest linewidth corresponds to the same scheme with 4% output reflector. For all schemes, the linewidth is proportional to the FBG bandwidth. The data show that the random laser exhibits much weaker broadening as compared with both traditional BDF laser configurations (with and without the PF). In the case of the narrowband FBG the random laser linewidth is below 0.15 nm at all powers being 2 times  smaller than that for the traditional laser in a similar configuration (see Fig. 4). The relative difference is even larger for the wideband FBG. Besides, the spectrum of random laser is quasi-continuous, while in traditional laser it consists of discrete modes.

To explain the uniquely weak power broadening in the random fiber laser scheme, we have developed the following analytical model.

### Analytical model

The main mechanism of spectral broadening in fiber lasers with negligible dispersion is defined by self-phase modulation (SPM)^24^. In our case, the laser generates near zero-dispersion wavelengths (1430 nm for the PF and 1480 nm for the BDF). The dispersion parameter *D* at 1420 nm amounts to −1.2 ps/nm·km and −3.5 ps/nm·km for the PF and BDF, respectively, so the influence of dispersion can be neglected, i.e. zero-dispersion assumption^24^ is applicable. As the pump wavelength (1310 nm) is not far enough, we also need to take into account the effect of pump-induced cross-phase modulation (XPM). The root-mean-square (RMS) width of the generated spectra 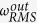 is defined by the expression (see Method section)


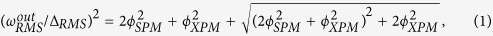


where Δ_RMS_ is the RMS width of the FBG spectra, *ϕ*_*SPM*_ and *ϕ*_*XPM*_ are the phase shifts induced by SPM and XPM, respectively. The nonlinear SPM phase in the cavity composed of passive and active fibers with lengths *L*_*p*_ and *L*_*a*_ respectively (Fig. 1), and mirrors with reflectance *R*_1_ ≪ 1 and *R*_2_ ~ 1 takes the form (see Method section)


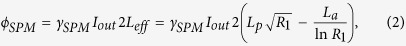


where *γ*_*SPM*_ ≈ 2.5 W^−1^ km^−1^ is the Kerr coefficient for a single-mode fiber at 1.42 μm, 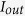 is the output power delivered from the low-reflectance (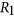 ≪ 1) output mirror, *L*_*eff*_ is the effective cavity length. The effective length is estimated as 200, 50 and 12 meters for the experimental schemes: PF-BDF with a point reflector (*R*_*1*_ = 4%), BDF with *R*_*1*_ = 4%, and PF-BDF with the RS (10^−5^), respectively. The nonlinear XPM phase is


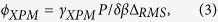


where *γ*_*XPM*_ = 2.*γ*_*SPM*_ ≈ 5.0 W^−1^ km^−1^ is the Kerr coefficient for the XPM process, *P* is the pump power, *δβ* ≈ 0.8 ps/m is the dispersion walk-off coefficient for the pump and signal waves.

**Figure 5:**
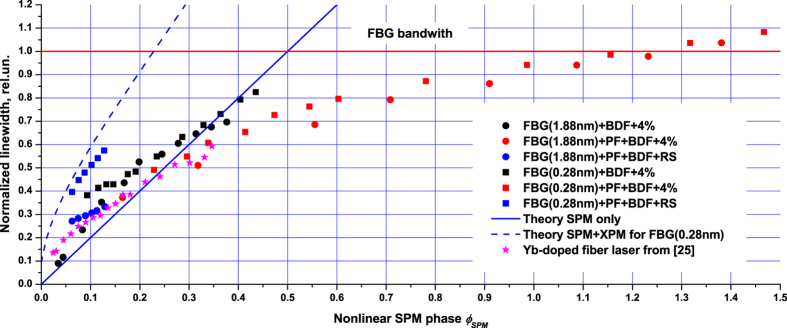
Comparison of the normalized lasing linewidth (related to the FWHM bandwidth of the FBG) for all schemes of the Bi-doped fiber laser as a function of the nonlinear phase (Eq. (2)). The data for the Yb-doped fiber laser from 25 are also added. The theoretical curves for SPM (solid line) and SPM combined with XPM in the case of narrowband FBG (dashedline) are given.

The data of Fig. 4a,b are replotted in Fig. 5 in normalized units: spectral linewidth related to the FBG bandwidth and output power reduced to the integral nonlinear phase (2). As reflection coefficient *R*_*1*_ is different in the studied configurations, the nonlinear phase is also different. For the sake of simplicity, we assume that the RS distributed feedback can be modeled as an effective point reflector with the reflectivity corresponding to the integral Rayleigh backscattering (*R*_*1*_ = 10^−5^) which is located at the end of the cavity. One can see from Fig. 5 that the normalized data for different schemes as well as for different FBGs are in good agreement with each other, except for the scheme with the narrowband FBG and RS. It should be noted that *ϕ*_*SPM*_ ≫ *ϕ*_*XPM*_for these schemes, so spectral broadening has linear character 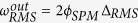 defined by the SPM effect. The experimental data at the nonlinear phase below 0.5 demonstrate the linear dependence with slope of about 2, which is in good agreement with the theoretical value. The deviations from the linear dependence are associated with limitations imposed by the FBG when the generation linewidth approaches FBG bandwidth and deviations from the Gaussian statistics of laser radiation at high nonlinearities, when expression (1) becomes incorrect.

In the case of the narrowband FBG and RS, the XPM effect becomes important, 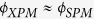, as a result spectral broadening has a different character. Figure 5 shows good agreement of the experimental data in this case with the theoretical curve involving XPM, which is calculated from Eqs (1, 2, 3) taking the measured laser to pump power ratio (Fig. 2a). It means that the nonlinear SPM phase (2) for the scheme with the RS and narrowband grating is so small that the effect of XPM makes a significant contribution to formation of the output spectrum. One can estimate the critical FBG reflection spectrum width at which the XPM phase becomes comparable with the SPM phase for the RS-based laser as 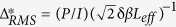 ≈ 0.15 ps^−1^, which corresponds to the FBG with ~0.37 nm width. It means that for narrower FBGs the XPM process becomes dominant. For the schemes with a point reflector, the effective cavity length is larger and the XPM contribution will be noticeable for FBG widths below 0.1 nm.

## Discussion

The performed analysis has shown that quite different power broadening curves (linewidth versus power) obtained for different cavity configurations (Fig. 4) merge together when re-drawn in normalized variables for the linewidth (related to the FBG bandwidth) and power (integrated over the cavity length with a nonlinear coefficient thus providing nonlinear phase incursion), see Fig. 5. So, the main broadening effect is self-phase modulation characterized by linear growth of the normalized linewidth versus the nonlinear phase, which is, in its turn, proportional to the output power. However, the proportionality coefficient is quite different for the random and normal-cavity configurations because of quite different longitudinal power distributions (see Methods section for details). For the laser with random distributed feedback the main part of power is concentrated near the output end thus resulting in the lowest nonlinear phase at the highest output power.

The effect of XPM induced by pump radiation also makes some contribution to the linewidth because of a low group velocity dispersion between the pump and Stokes waves, but it is negligibly small in the schemes with broadband FBGs characterized by the broad SPM-induced linewidth (in absolute values), and rather weak in the schemes with narrowband FBG and 4% reflector. It becomes significant only in the scheme with narrowband FBG and extremely weak RS feedback (10^−5^), adding a specific nonlinear component into the linewidth-power dependence.

It is also interesting that the data for Yb-doped fiber laser (YDFL) linewidth broadening^25^ plotted in the same plane of the normalized linewidth and nonlinear phase fit quite well with both the Bi-doped fiber laser data and the developed theory (Fig. 5). The XPM effect in this case is relatively small because of a large group velocity dispersion of the pump and laser waves, in spite of the narrow YDFL spectrum defined by the narrowband output FBG (0.067 nm). There are also other distinctions between the YDFL and studied Bi-doped laser configurations. The active fiber as well as the cavity as a whole is much longer in Bi-doped lasers, whereas the output power is sixfold higher for the Yb-doped fiber laser. Nevertheless, the values of the nonlinear phase have the same order for both schemes (~0.5) as a result of power integration over the length. In spite of a much narrower laser linewidth of the Yb-doped laser (especially in comparison with the case of the Bi laser with 1.88-nm FBG) the values normalized to the FBG bandwidth behave quite similarly in accordance with the SPM theory (Fig. 5).

It is also important to discuss the role of passive fiber. As mentioned above, the laser generation becomes unstable when passive fiber is located after BDF. It means that the Rayleigh backscattering associated with passive fiber is not a key point in the stabilization of laser generation. Though the integral reflectivity of PF (6.2·10^−6^) is comparable with that for BDF (3.8·10^−6^), the fraction of signal power reflected from BDF is much greater than that from passive fiber because the signal power reaches its maximum in the active fiber when it is located at the output end (see Methods section). Thus, in the studied configuration the BDF has a decisive contribution to the formation of feedback, as well as to the amplification of signal, just like in other random lasers including random fiber lasers^1^,^2^,^3^,^4^,^5^,^6^,^7^,^8^. However, a passive fiber located inside the cavity in front of BDF plays an important role in the stabilization of laser generation. We believe that the role of passive fiber consists in the spectral broadening due to cross-phase modulation induced by the unabsorbed pump, which suppresses Brillouin scattering thus resulting in the stabilization of random lasing. Indeed, the effect of cross-phase modulation is able to suppress the Brillouin instability as soon as the spectral broadening exceeds the Brillouin gain bandwidth^26^. When the passive fiber is located after the Bi-doped fiber the XPM-induced spectral broadening is much less than that for the studied scheme, because of the pump power absorption in Bi-doped fiber. Experiments with different passive fibers having similar Rayleigh backscattering coefficients also confirm no significant influence of Rayleigh scattering in passive fiber on the process of lasing stabilization.

In conclusion, we have demonstrated the first RS-based random fiber laser based on a Bi-doped active fiber and compared it with the laser having a normal cavity formed by normal cleaving of the output fiber end instead of angled cleaving, with other components left unchanged. Though the power characteristics of these configurations are similar, the linewidth in the case of random laser appears to be much narrower. The developed theory of nonlinear broadening explains this effect by a specific power distribution in the case of extremely weak RS-based feedback resulting in a small value of the SPM-induced nonlinear phase at high output powers. Moreover, a comparison of power broadening for different experimental schemes in normalized units for the linewidth and power (divided by the FBG bandwidth and multiplied by the effective length, respectively) reveals very good agreement between them. The only deviation is observed in the case of random fiber laser with the narrowband FBG, when the XPM contribution becomes comparable with the SPM width thus resulting in a considerable nonlinear addition in the linewidth-power dependence. Thus, the developed unified SPM + XPM theory describes all configurations. The demonstrated narrowband Bi-doped random fiber laser is also attractive for applications such as sensing and telecommunications (e.g. as a modeless pump source for distributed Raman amplifiers^27^), as well as bio-imaging taking into account a possibility of its efficient conversion into the visible range. Note that lasers based on Bi-doped fibers with different host compositions and appropriate pumping can generate in an extremely broad range of wavelengths, 1150–1775 nm^28^,^29^.

## Methods

### Analytical expression for spectral broadening due to the combined effect of self- and cross- phase modulation

Let us introduce a correlation function for the signal (*s*) and pump (*p*) waves, similar to^24^:


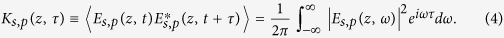


Here *E*_*s*_,_*p*_(*z*, *t*)/*E*_*s*_,_*p*_(*z*, *ω*) is the electrical field in the time/frequency domain for the signal (s) and pump (p) waves, respectively, the angular brackets denote averaging over the Gaussian statistics of the laser field. The values of the correlation function and of its first and second derivatives at *τ* = 0 define the mean value of intensity 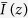, the mean value of frequency 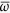 and the root-mean-square (RMS) frequency *ω*_*RMS*_ correspondingly:


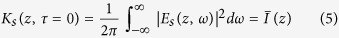



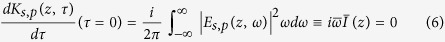



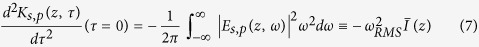


The correlation function at the output end (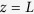) for the forward signal wave in the case of a small XPM-induced phase shift has the form similar to that for SPM in the presence of gain^30^,^31^:


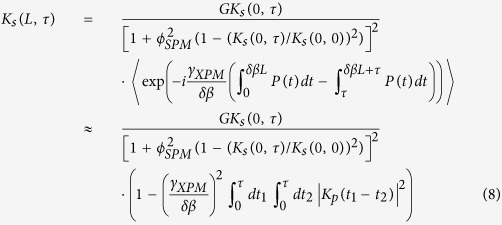


where *G* is the integral gain, 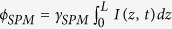 is the nonlinear phase, *γ*_*SPM*_ and *γ*_*XPM*_ are the Kerr coefficients for SPM and XPM, respectively, *δβ* is the dispersion walk-off coefficient for the pump and signal waves, I(*z, t*) = |*E*_*s*_(*z*, *t*)|^2^ and *P*(*z*, *t*) = |*E*_*p*_(*z*, *t*)|^2^ are the signal pump power, relatively. The first and second factors in Eq. (8) are related to the SPM and XPM processes, respectively.

The correlation functions for the backward waves have a similar form, but without the XPM factor. As a result, the correlation function over one roundtrip in the cavity takes the form


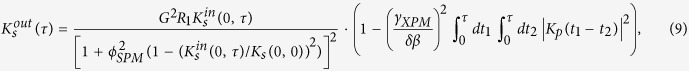


where *R*_1_ is the effective reflectance of broadband output mirror at *z* = *L*. It should be noted that the SPM effect plays a role for forward and backward waves. After double differentiation of Eq. (9) one can obtain the relation between the RMS frequencies of the input and output correlation function:


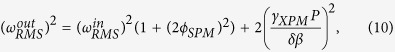


where *P*(*z*, *t*) = *P*_+_(*z*, *t*) ≡ *P* as only a co-propagating pump wave induces XPM, whereas *I*(*z*, *t*) = *I*_+_(*z*, *t*) + *I*_−_(*z*, *t*). To find the RMS frequency 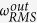 for the output correlation function, an equation describing FBG reflection is required. Let us assume that all spectral profiles have the Gaussian shape, i.e. 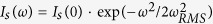 for the signal and 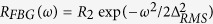 for the FBG reflection coefficient. In this case one can derive the relation


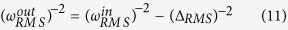


Equations (10) and (11) form a closed system. As a result, the normalized spectral linewidth takes the form


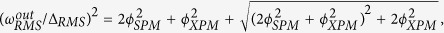


where *ϕ*_*XPM*_ = *γ*_*XPM*_*P*/*δβ*Δ_*RMS*_ is the nonlinear phase shift due to the XPM process. Without XPM the spectral broadening is a linear function of the SPM-induced phase, 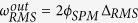, similar to^25^. It should be noted that the constant in ref. 25 is different due to another spectral contour supposed (hyperbolic secant), the ratio between the RMS and FWHM widths is also dependent on the spectral shape (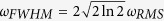 for the Gaussian shape of spectra). The FWHM width of spectra in wavelength units (as measured in the experiment) has the form 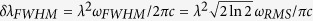.

So, taking XPM into account results in an addition, which varies with power nonlinearly even at a linear ratio between the pump and laser power. The particular value of the coefficient between the linewidth and output power depends also on the longitudinal power distribution defining the integral nonlinear phase, which, in its turn, is different in different cavity configurations.

### Longitudinal power distribution and nonlinear phase

As follows from the previous section, the nonlinear phase strongly depends on the signal power distribution in the cavity for the forward *I*_+_(*z*, *t*) and backward *I*_−_(*z*, *t*) waves. Let us consider the cavity composed from passive and active fibers with length *L*_*p*_ and *L*_*a*_, respectively, and mirrors at the opposite cavity ends with reflectivity *R*_1_ and *R*_2_. Let us also assume that the power in passive fiber is independent of longitudinal coordinate *z* and the power in active fiber is exponentially growing. From the condition of equality between loss and gain over a roundtrip, we obtain the power distribution


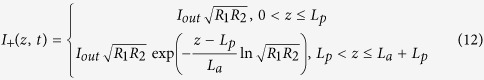



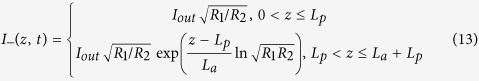


where *I*_*out*_ is the output power delivered from the output mirror with reflection coefficient *R*_1_. So, the highest power is reached for forward wave in the active fiber output. Herewith, the nonlinear phase in case of *R*_1_ ≪ 1 and *R*_*2*_ ~ 1 takes the form


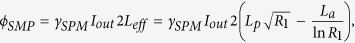


where *L*_*eff*_ is the effective cavity length. Thus, the ratio between the nonlinear phase and output laser power is strongly dependent on the output reflection coefficient *R*_*1*_, with its minimum value being reached in the case of an extremely weak RS-based feedback.

## Additional Information

**How to cite this article**: Lobach, I. A. *et al*. Narrowband random lasing in a Bismuth-doped active fiber. *Sci. Rep*. **6**, 30083; doi: 10.1038/srep30083 (2016).
